# Nucleic acid synthesis screening at an inflection point: U.S. policy momentum and global implications

**DOI:** 10.3389/fbioe.2026.1843263

**Published:** 2026-10-08

**Authors:** Ishan Mehta, Melissa Hopkins

**Affiliations:** Department of Environmental Health and Engineering, Johns Hopkins Center for Health Security, Bloomberg School of Public Health, Johns Hopkins University, Baltimore, MD, United States

**Keywords:** artificial intelligence, biosecurity, governance, politics, public policy, regulation, synthetic biology

## Abstract

This article examines the positioning of nucleic acid synthesis screening (NASS) policy within the current U.S. political landscape, tracing its evolution from voluntary industry practices to executive and legislative action across administrations. We analyze the sources of NASS’s political momentum, including the rare convergence of bipartisan national security imperatives, industry support for codified screening requirements, and biosecurity-related incidents. We assess key policy challenges, including navigating jurisdictional limitations and congressional gridlock, the risk of regulatory capture, and ensuring regulatory frameworks remain adaptive to evolving technologies. We situate U.S. efforts within an international context, examining parallel initiatives in the United Kingdom, the European Union, and New Zealand, noting how U.S. market-access requirements may promote *de facto* global screening adoption through economic incentives rather than multilateral diplomacy. We recommend specific policy instruments, such as expanding the U.S.-based third-party screening industry, leveraging federal procurement requirements, aligning with ISO standards, and building on NIST’s maturing capability-based benchmarks, to establish enforceable domestic requirements while extending global reach.

## 1 Introduction: the case for nucleic acid synthesis screening

Nucleic acid synthesis is inherently dual use, in that the same capabilities that enable researchers to develop vaccines and therapeutics could be exploited to synthesize dangerous pathogens or give physical form to harmful biological agents designed with artificial intelligence (AI). High-profile episodes such as the 2006 purchase of a smallpox DNA fragment from a commercial supplier without adequate vetting (Randerson, 2006) and the reconstruction of the 1918 pandemic influenza virus from synthetic DNA (Taubenberger et al., 2007) confirmed that existing biosecurity safeguards could be circumvented (Minshull and Wagner, 2009). In response, the International Gene Synthesis Consortium (IGSC) (Synthesis, 2020) championed customer and order screening protocols (Johns Hopkins Center for Health Security, 2025), but for decades these efforts remained largely voluntary, with federal guidance published only twice over a 10-year gap, and no binding enforcement mechanism (Administration for Strategic Preparedness and Response (2023)).

That is now changing. Nucleic acid synthesis screening (NASS) is gaining significant political momentum in the United States, driven by Executive Branch actions across administrations, such as the Biden Administration’s Framework for Nucleic Acid Synthesis Screening (Framework)^1^, which conditioned federal funding on procurement from compliant providers of synthetic nucleic acids (providers)^2^, and a Trump Administration executive order emphasizing enforcement and expanded coverage (The White House, 2025b). Building on executive action, there have been several bipartisan congressional bills the National Security Commission on Emerging Biotechnology’s recommendations on NASS (U.S. Congress. H.R. 3029, 2025), and industry self-regulation through the IGSC and the Sequence Biosecurity Risk Consortium (SBRC) (SBRC, 2025). Industry actors have broadly supported codifying screening requirements. While the details of such codifications matter (IGSC, 2026), it is relatively rare to see industry players supportive of requirements. This political and industry momentum coincides with heightened urgency: advances in generative AI now touch nearly every step of the bioweapons development process, from ideation (OpenAI, 2024) and pathogen design (King et al., 2025) to testing (Anthropic, 2026), materials acquisition (Brady et al., 2026), and dispersal (Mouton et al., 2024). Advances in these capabilities through AI are compounded by the rise of automated laboratories and agentic AI (Tobias and Adam, 2025). Despite such new technologies and potential physical chokepoints, researchers maintain that nucleic acid synthesis—the point at which a digital sequence becomes physical genetic material—remains the key chokepoint at which misuse can be intercepted (Schmitt and Monrad, 2026).

This article examines NASS policy within the current U.S. political landscape, shaped by the convergence of bipartisan national security imperatives, industry support for codified screening requirements, and biosecurity-related incidents. We trace the policy evolution from early voluntary screening through current executive and legislative actions, assess challenges including congressional gridlock, budgetary constraints, jurisdictional limitations, and technological adaptability, and situate U.S. efforts alongside parallel initiatives in the United Kingdom, the European Union, and New Zealand. We argue that NASS represents a rare point of bipartisan alignment—one that simultaneously strengthens national security and cultivates a growing domestic industry—and that the United States is uniquely positioned to lead in establishing domestic requirements that, through market-access incentives, promote screening adoption globally.

## 2 Current policy landscape

U.S. institutional concerns and attention to synthetic biology began in the early 2000s with the high-profile demonstrations of reconstructed pathogens from sequence data (Kelle, 2009). The cross-disciplinary National Science Advisory Board on Biosecurity (NSABB), established by the U.S. Department of Health and Human Services (HHS), in 2006 recommended a focus on the transition point between the digital and physical where synthetic DNA is ordered and fulfilled (U.S. Department of Health and Human Services, 2010).

Commercial providers began adopting order-screening practices for sequences and customers (Know-Your-Customer, or KYC, requirements) in the late 2000s, before screening was uniformly expected across the market, driven both by biosecurity risk concerns and by reputational and regulatory considerations.

In 2010, HHS introduced the “Screening Framework Guidance for Providers of Synthetic Double-Stranded DNA”, the first voluntary guidance that emphasized risk-based screening and sought to standardize industry practices on customer and sequence screening (US Department of Health and Human Services, 2010). Industry harmonization also coalesced around the IGSC, which standardized screening protocols to reduce venue shopping, where an actor may attempt to acquire material from multiple providers to find one willing to sell material without screening, and established shared baselines for match thresholds, escalation, and recordkeeping (International Gene Synthesis Consortium, 2024).

However, technology and markets, especially shorter oligonucleotides, continued shifting and affecting the impact of the guidance and industry efforts. In 2023, President Biden signed Executive Order 14110 (The White House. Executive Order 14110, 2023), which followed HHS beginning to update its 2010 guidance the previous year to broaden and standardize screening across the existing ecosystem, as well as address rapid technological progress in the market (U.S. Department of Health and Human Services, 2023a). Most importantly, the updated guidance was built upon by the Framework a year later. The Framework issued a federal funding requirement to ensure that recipients of federal funds procure synthetic nucleic acids only from providers that adhere to the Framework (The White House, 2024). By 2024, major providers commonly screened orders, but smaller providers remained inconsistent^1^. Industry coalesced around an ISO standard for the production and quality control of synthetic nucleic acids (International Organization for Standardization ISO, 2020) with companies like Twist and Ginkgo also endorsing NASS bills aligned with the Framework (Office of Senator Tom Cotton, 2026).

Political change brought new dynamics but kept NASS a priority: the Trump Administration withdrew E.O. 14110 (The White House, 2025a) but issued a new Executive Order (E.O. 14292), requiring an updated or replacement Framework with stronger enforcement and consideration of private sector requirements, requiring NASS for non-federally funded entities and covering the entire market (The White House, 2025b). The updated Framework is still pending as of March 2026.

At the same time, there is growing momentum in the U.S. Congress for NASS. As Table 1 illustrates, NASS policy activity has accelerated dramatically, growing from just 3 federal actions across the 12 years between 2006 and 2018 to 18 actions in the 4 years since 2022 alone, reflecting a broadening base of support across both parties, multiple agencies, both chambers of Congress, and independent commissions. Most promisingly, in 2026, the first bipartisan bill to require private sector screening was introduced by Senators Cotton and Klobuchar. The bill seeks to create alignment with the provisions of E.O. 14292 and proposes a concrete enforcement mechanism for NASS (US Congress S 3741, 2026). There has also been momentum building at the state level with bills introduced in the New York and California legislatures that require NASS (Legislative Session, 2025; California State Assembly, 2026).

**TABLE 1:** U.S. NASS policy actions.

Date	Document	Jurisdiction	Type	Issuing body/sponsor
Dec. 2006	Addressing Biosecurity Concerns Related to Synthetic Biology (National Science Advisory Board, 2006)	Federal	Report	National Science Advisory Board for Biosecurity
Oct. 13, 2010	Screening Framework Guidance for Providers of Synthetic Double-Stranded DNA (U.S. Department of Health and Human Services, 2010)	Federal	Guidance/Framework	Department of Health and Human Services
Sep. 18, 2018	National Biodefense Strategy (The White House, 2018)	Federal	Report	The White House
Aug. 26, 2022	AB-1963 — California Educational Code §§ 66,360–66361 (gene synthesis providers; CSU/UC) (California Educational, 2022)	California	Statute (in effect)	Calif. Assemblymember Rudy Salas
Sep. 12, 2022	Advancing Biotechnology and Biomanufacturing Innovation for a Sustainable, Safe, and Secure American Bioeconomy (The White House. Executive Order 14081, 2022)	Federal	Executive order	The White House
Oct. 18, 2022	National Biodefense Strategy & Implementation Plan 2022 (The White House, 2022)	Federal	Report	The White House
Oct. 25, 2022	Eshoo Urges NSA and OSTP to Address Biosecurity Risks Caused by AI (Letter by Representative Anna G, 2022)	Federal	Letter	Rep. Anna Eshoo
Apr. 26, 2023	Guidance Memo 23–02: Guidance on Purchasing Gene Synthesis Equipment or Products (University of California and Research Policy Analysis and Coordination RPAC, 2023)	California (University of California)	Guidance memo	University of California
July 18, 2023	S. 2356 (118th), Gene Synthesis Safety and Security Act (U.S. Congress. S. 2356, 2023)	Federal	Bill	Sens. John Hickenlooper and Ted Budd
July 21, 2023	H.R. 4702/S. 2400 (118th), Securing Gene Synthesis Act (U.S. Congress. H.R. 4702, 2023)	Federal	Bill	Rep. Anna Eshoo and Sen. Ed Markey
Aug. 17, 2023	Biodefense Posture Review (United States Department of Defense, 2023)	Federal	Strategy	U.S. Department of Defense
Oct. 30, 2023	Safe, Secure, and Trustworthy Development and Use of Artificial Intelligence (The White House. Executive Order 14110, 2023)	Federal	Executive Order	The White House
Feb. 16, 2024	NIST, Nonprofit Research Consortium to Develop Safety Tools for Synthetic Biology to Defend Against Potential Misuse of AI (National Institute of Standards and Technology, 2024a)	Federal	Announcement	U.S. Department of Commerce/NIST
Mar. 3, 2024	Artificial Intelligence: Roadmap 2024 (U.S. Department of Homeland Security, 2024)	Federal	Strategy/roadmap	U.S. Department of Homeland Security
Apr. 10, 2024	Gene Synthesis Screening Letter (Office of Representative Julia Brownley, 2024)	Federal	Letter to Executive Branch	Rep. Julia Brownley et al.
Apr. 29, 2024	Framework for Nucleic Acid Synthesis Screening (U.S. Department of Health and Human Services, 2023b)	Federal	Framework	The White House
Apr. 2024	Gene Synthesis Security (National Security Commission on Emerging Biotechnology, 2024)	Federal	Issue Paper	National Security Commission on Emerging Biotechnology
Aug. 16, 2024	Biosecurity for Synthetic Nucleic Acid Sequences (National Institute of Standards and Technology, 2024b)	Federal	Publication	National Institute of Standards and Technology (NIST)
Aug. 29, 2024	H.R. 9194 Nucleic Acid Standards for Biosecurity Act (U.S. Congress. H.R. 9194, 2024)	Federal	Press release	Reps. Yadira Caraveo and Rich McCormick
Jan 27, 2025	Assembly Bill A3283A (Legislative Session, 2025)	New York	Bill	Assemblymember Alex Bores
Apr. 8, 2025	Final Report (National Security Commission on Emerging Biotechnology, 2025)	Federal	Commission report	National Security Commission on Emerging Biotechnology
Apr. 29, 2025	H.R. 3029 Nucleic Acid Standards for Biosecurity Act (U.S. Congress. H.R. 3029, 2025)	Federal	Bill	Reps. Andrea Salinas and Richard McCormick
May 5, 2025	Improving the Safety and Security of Biological Research (The White House, 2025b)	Federal	Executive order	The White House
Jan. 29, 2026	S. 3741 Biosecurity Modernization and Innovation Act (U.S. Congress. S. 3741, 2026)	Federal	Bill	Senators Tom Cotton and Amy Klobuchar
Feb. 11, 2026	Assembly Bill 1864 (California State Assembly, 2026)	California	Bill	Assemblymember Marc Berman

The acceleration of NASS policy in the United States can be understood through Kingdon’s multiple streams framework, which illuminates how the convergence of problem recognition, policy solutions, and political conditions created the current window of opportunity for regulatory action (Kingdon, 1984). The problem of NASS screening gaps gained recognition through pathogen reconstruction episodes, the coronavirus pandemic making biosecurity salient, and AI-enabled bioweapon risks detailed above. Policy solutions matured as voluntary industry protocols gave way to federal guidance and executive orders. Politics aligned as U.S. intelligence begun to affirmatively state the existence of active offensive bioweapons programs (DNI, 2021), national security imperatives such as the privately funded bipartisan Biodefense Commission (Bipartisan Commission on Biodefense, 2014), the National Biodefense Strategy (The White House, 2022) and the Biodefense Posture Review (United States Department of Defense, 2023), and the uncommon phenomenon of industry players being supportive of regulatory requirements created favorable conditions across administrations. The resulting policy window is evidenced by the acceleration of NASS activity shown in Tables 1 and 2.

**TABLE 2:** International NASS policies.

Date	Country	Nucleic acid synthesis screening policy	Status	Summary
Oct. 8, 2024	United Kingdom	UK Screening Guidance on Synthetic Nucleic Acids for Users and Providers	Published	Voluntary guidance for sequence and customer screening with risk-based escalation (UK Department for Science and Innovation and Technology DSIT, 2024)
Oct. 10, 2025	New Zealand	Gene Technology Bill	Under Consideration	Mandate for screening, directs regulators to establish framework with procedural details (Gene, 2024)
Dec. 16, 2025	European Union	EU Biotechnology and Biomanufacturing Act (Proposal)	Under Consideration	Mandate for screening for all businesses that sell or manufacture nucleic acids in the European Union. Enforcement distributed to member states (European Commission, 2022)

Matching U.S. momentum, Table 2 shows international momentum in the past few years as other jurisdictions have tackled NASS in varying capacities. The European Union’s proposed Biotech Act includes NASS provisions requiring member states to conduct risk-based audits of providers and establishes penalties up to 5% of annual global revenue for non-compliance; adoption is expected in 2027 (European Commission, 2022). New Zealand’s Gene Technology bill would create private sector requirements but has stalled over controversial GMO provisions (Gene, 2024; Meyer, 2026). The United Kingdom issued comprehensive guidance for nucleic acid providers and customers, similar to the 2023 HHS guidance, but lacks enforcement mechanisms comparable to the U.S. federal funding requirement in the Framework (UK Department for Science, Innovation and Technology (DSIT), 2024).

The adoption pattern reflects two factors: informal governance networks among officials in these jurisdictions^3^, and relative market size. These factors explain why China and India, with large markets but weaker U.S. diplomatic engagement, lack NASS policies, while New Zealand acted despite its smaller market due to network influence.

Divergent governance approaches also reflect broader tech-regulation postures. The UK has mirrored U.S. approaches, such as the UK taking the US’ lead in its establishment and rebranding of the Artificial Intelligence Safety (now “Security”) Institute (Gold, 2025) and its refusal to sign the Paris AI Accord (Firstpost, 2025). In contrast, the EU and New Zealand have historically favored stronger technology regulation (European Parliament and Council of the European Union, 2016).

Despite limited national mandates, industry consortia and international cooperation create indirect pressure for screening adoption. China, the largest market without national NASS guidance, has major providers like BGI and Tsingke Biotech participating in IGSC and attesting to screening compliance (International Gene Synthesis Consortium, 2025). Additionally, provisions like the U.S. federal funding requirement incentivize international providers to adopt screening to access federally funded U.S. customers, promoting *de facto* global harmonization.

## 3 Challenges

While the domestic and international momentum described above is encouraging, significant obstacles remain. U.S. congressional dysfunction and the current political climate make it difficult for any policy area to make progress, and NASS is no exception. As noted earlier, the change in administrations was a setback to NASS and biosecurity when President Trump withdrew E.O. 14110. Since E.O. 14110 established the creation of the Framework, this withdrawal of the E.O. called its standing into question. HHS has had important biosecurity policy work stalled because of reductions in workforce and budgets, Congress has enacted fewer bills than ever before, and partisan and jurisdictional conflicts jeopardize even bipartisan bills from making progress (Kim and Wu, 2025; Wallach, 2025).

Additionally, as U.S.-China competition progresses, a heightened mistrust can inhibit cross-border information sharing about suspicious customers or orders, even though such coordination is often essential for escalation, referral, and detection of repeat or distributed procurement attempts. A recent incident when federal agents discovered an illegal lab near Las Vegas run by Chinese nationals exemplified some of these fears and led to calls for tighter biosecurity measures, especially for providers from China (Fox News, 2024). Despite this concern, the U.S. maintains leadership in terms of screening providers as well as policy. The IGSC is led by U.S.-based providers, but the largest Chinese providers, such as BGI, have also joined and attested to screening orders based on IGSC standards. This demonstrates that despite challenges in information sharing between the U.S. and China, agreement can still be met through industry and security requirements that benefit both countries.

Another challenge is creating policy solutions that are adaptable enough to change in technology and the market. For example, as AI tools make it easier to generate sequences to evade NASS, policies focused on list-based screening may quickly become obsolete (Wittmann et al., 2025).

The nucleic acid synthesis industry is also subject to import and export control regulations that intersect with NASS policy objectives. The U.S. Department of Commerce Export Administration Regulations (EAR) and the select agent regulations impose restrictions on the transfer of certain biological materials and related technologies across border (CFR, 2020). These regulatory mechanisms are another layer of governance that NASS policies must navigate and complement. However, export controls have historically focused on list-based products, such as lists of sequences or select agents, rather than a risk-based framework tailored to the synthesis process itself, potentially leaving a disjoint governance regime for providers to navigate.

Finally, as with any regulatory policy, there is also a risk of cementing incumbents through regulatory capture. Because larger industry incumbents have a higher capacity for compliance and thus stand to benefit from NASS regulations, policymakers must ensure that as the market matures and dynamics change, regulations do not just favor larger providers and leave sufficient room for innovative solutions and smaller industry players.

## 4 Actionable recommendations

Despite the above challenges, there is a burgeoning opportunity for the U.S. to reduce biosecurity risks from synthetic nucleic acids and establish leadership in a growing market. As mentioned above, nearly all of the screening companies responsible for supplying screening services to providers or manufacturers of nucleic acid synthesis equipment (manufacturers of, e.g., benchtop devices) are U.S.-based companies (Faster Capital, 2024). Due to this and other factors, domestic regulation alone could reduce a significant amount of risk while bolstering the U.S. bioeconomy. The bipartisan alignment in achieving these dual goals may be capable of cutting through congressional gridlock. Leveraging U.S. market dominance, growing the domestic screening industry, and building on maturing technical standards together address the additional challenges identified above.

The U.S. nucleic acid synthesis purchasing market is the largest in the world, holding around 30%–40% of synthetic DNA purchasing worldwide, depending on the specific market segments included, with North America as a whole closer to 40%–55% (Mordor Intelligence, 2025). Most purchases stem from pharmaceutical companies, academic institutions, and contract research organizations/manufacturing organizations (Precedence Research, 2025). Increasingly, many purchases are for oligonucleotides (nucleic acid sequences shorter than gene length), which capture an even greater U.S. market share (Mordor Intelligence, 2025). As noted in the discussion of *de facto* global harmonization above, any provider or manufacturer seeking access to the U.S. market would need to comply with domestic screening requirements regardless of where they are based. This means the effective reach of U.S. regulation extends beyond the 30%–40% domestic market share alone, plausibly mitigating risk from roughly half of global orders (Casagrande, 2025). Importantly, this market-access approach also mitigates cross-border information-sharing challenges outlined above: compliance becomes a precondition for market participation, shifting the coordination mechanism from diplomacy to economic incentive.

In addition to targeting U.S. domestic NASS, the current market of NASS third-party services should be expanded domestically and exported globally. Third-party screening services are independent organizations that provide sequence screening and customer verification tools to providers (as distinct from providers conducting screening in-house), nearly all of which are U.S.-based (Johns Hopkins Center for Health Security, 2026). Targeted policy instruments can accelerate this expansion: tax incentives for domestic screening service providers, export credit and trade financing support for companies seeking to serve international markets, and NIST-backed certification programs that give compliant providers a recognized quality mark for global use (Faster Capital, 2024). These instruments, combined with procurement requirements such as those included in the Framework, can further grow the domestic market for third-party screening services. A useful precedent is the U.S. third-party pharmaceutical testing industry, where FDA’s stringent standards created early demand for compliance services and gave U.S. firms a self-reinforcing first-mover advantage globally. The lesson: strict domestic standards, paired with a large purchasing market and first-mover advantage, can seed an export industry in compliance services. Growing this market also helps guard against the regulatory capture risks identified above, since a more competitive ecosystem supported by clear, publicly accessible National Institute of Standards and Technology (NIST) standards lowers barriers to entry for smaller providers rather than requiring them to build costly in-house capabilities.

While requiring third-party screening too quickly risks constricting the market, well-implemented testing frameworks—introduced once validated measurement methods and sufficient market infrastructure are in place—historically tended to have market-expanding effects. Screening requirements are only as effective as the methods available to verify that screening tools actually work—that is, methods for measuring what a screening tool can detect (its capabilities) and for evaluating whether a given sequence poses a biosecurity concern (risk assessment) (Johns Hopkins Center for Health Security, 2025). Without such validated measurement methods, screening mandates risk becoming compliance exercises without substantive security value. Reliable methods for this purpose are now emerging. NIST has been developing exactly these kinds of capability benchmarks and risk assessment tools for several years, with encouraging results (Laird et al., 2025). Crucially, this capability-based approach also addresses a challenge identified earlier in this article: that AI tools could be used to generate sequences designed to evade traditional list-based screening. Standards rooted in functional capability rather than static reference databases are inherently more resilient to such evasion techniques and offer a way to future-proof against technological changes. Together with the technical progress that NIST and others have made in capability assessments, NASS has reached sufficient maturity for a transition to broader domestic requirements that the rest of the industry, including smaller providers, would be capable of adopting without the market-constricting effects of premature regulation.

Federal procurement requirements can drive global screening adoption through three reinforcing channels. First, they function as a market-access gate: any international provider seeking to serve U.S.-funded researchers must meet U.S. screening standards, effectively extending domestic requirements extraterritorially. Second, this creates strong incentives for alignment with ISO 20688–2:2024 (International Organization for Standardization ISO, 2024) (containing the international standard for synthesis screening), because providers benefit from a single recognized certification across jurisdictions. As U.S. procurement rules build upon ISO standards, NIST is well positioned to shape international standards from within, given its role administering and chairing the U.S. Technical Advisory Group to ISO/TC 276 (Biotechnology) (National Institute of Standards and Technology, 2022). Third, trade agreement frameworks provide additional leverage: screening requirements can be incorporated into World Trade Organization (WTO) Technical Barriers to Trade notifications, ensuring U.S. standards are transparent and defensible (World Trade Organization, 1994). Bilateral and plurilateral trade negotiations—such as those under the U.S.-EU Trade and Technology Council (U.S. Department of State, 2019) or Indo-Pacific Economic Framework (U.S. Department of Commerce, 2025)—offer venues for mutual recognition of screening certifications. Together, procurement requirements set the floor, ISO standards provide common technical language, and trade agreements formalize mutual recognition—promoting *de facto* global harmonization without a single multilateral treaty (Bradford, 2012). Policymakers should consider harmonizing NASS policy with existing import/export controls to avoid duplicative compliance burdens.

Together these strategies position the U.S. to address the biosecurity risks of nucleic acid synthesis while strengthening a domestic industry in which it already holds a significant advantage. The window for action is narrow: political momentum, technical readiness, and market position are currently aligned, but none of these conditions is guaranteed to persist. Establishing enforceable domestic requirements now, while growing the third-party screening ecosystem and leveraging market access to promote global adoption, would represent a pragmatic and consequential step toward reducing one of the most significant biosecurity risks of the coming decade.

## 5 Conclusion

NASS policy represents a rare convergence of bipartisan political will, industry support, and technical readiness. The acceleration of policy activity since 2022, the maturation of capability-based screening standards, and the U.S.'s dominant position in global nucleic acid purchasing together create a window of opportunity that may not remain open indefinitely. By establishing enforceable domestic requirements and growing the U.S.-based third-party screening industry, the U.S. can simultaneously reduce biosecurity risk and promote screening adoption globally through market-access incentives. Acting while political momentum and technical capacity converge would represent a pragmatic and consequential step toward addressing one of the most significant biosecurity challenges of the coming decade.

## Footnotes

1 Organizations (2026). Evidence to Support this Statement is Derived from Non-public Research Carried out by Several Organizations and Shared Directly with the Authors.

2 Providers (2020). The Authors Use “Providers” in This Paper to Refer Generally to Providers of Synthetic Nucleic Acids Unless Otherwise Stated, Such as for Screeners Who Provide Screening Services to Providers.

3 Anecdotal (2025). Anecdotal Evidence Based on Personal Involvement in Several of These Discussions.
